# Effective reinforcements for thermoplastics based on carbon nanotubes of oil fly ash

**DOI:** 10.1038/s41598-019-56777-1

**Published:** 2019-12-30

**Authors:** Numan Salah, Abdulrahman Muhammad Alfawzan, Abdu Saeed, Ahmed Alshahrie, Waleed Allafi

**Affiliations:** 1 Centre of Nanotechnology, King Abdulaziz University, Jeddah, Saudi Arabia; 2 SABIC Plastic Application Development Center, Riyadh, 11551 Saudi Arabia; 3 Department of Physics, Faculty of Sciences, King Abdulaziz University, Jeddah, Saudi Arabia

**Keywords:** Engineering, Materials science, Nanoscience and technology

## Abstract

Carbon nanotubes (CNTs) are widely investigated for preparing polymer nanocomposites, owing to their unique mechanical properties. However, dispersing CNTs uniformly in a polymer matrix and controlling their entanglement/agglomeration are still big technical challenges to be overcome. The costs of their raw materials and production are also still high. In this work, we propose the use of CNTs grown on oil fly ash to solve these issues. The CNTs of oil fly ash were evaluated as reinforcing materials for some common thermoplastics. High-density polyethylene (HDPE) was mainly reinforced with various weight fractions of CNTs. Xylene was used as a solvent to dissolve HDPE and to uniformly disperse the CNTs. Significantly enhanced mechanical properties of HDPE reinforced at a low weight fraction of these CNTs (1–2 wt.%), mainly the tensile strength, Young’s modulus, stiffness, and hardness, were observed. The tensile strength and Young’s modulus were enhanced by ~20 and 38%, respectively. Moreover, the nanoindentation results were found to be in support to these findings. Polycarbonate, polypropylene, and polystyrene were also preliminarily evaluated after reinforcement with 1 wt.% CNTs. The tensile strength and Young’s Modulus were increased after reinforcement with CNTs. These results demonstrate that the CNTs of the solid waste, oil fly ash, might serve as an appropriate reinforcing material for different thermoplastics polymers.

## Introduction

Polymer nanocomposites are promising for application in various products like automobile, ships, aircrafts, furniture, pipes, constructing materials, industrial parts, and food packaging^1^. Different nanostructures have been synthesised and investigated as polymer fillers to enhance certain properties of polymers^2,3^. Among such structures, carbon nanotubes (CNTs) are found to have superior mechanical and electrical properties^2,4,5^. CNTs are either single-walled or are formed from multi-walled graphene sheets. Polymer nanocomposites based on CNTs are in high demand because they are lightweight, and have excellent mechanical strength and good electrical conductivity^4,5^. This has encouraged extensive investigation of polymer nanocomposites based on CNTs.

Although commercially available CNTs are under continuous development and their production rate has been increasing every year, they still face some major problems. Their price is higher than those of other commonly used/recommended fillers, which is one of the major challenges hindering the advancement of CNTs and CNT composite industry^6^. Other technical problems that still remain unsolved are agglomeration, dispersion, and interfacial bonding of CNTs, which hinder complete utilisation of the properties of the CNTs in composites^4^. Further, the degree of dispersion of CNTs in a polymer matrix is quite important, as it correlates with the mechanical performance of the polymer-CNT nanocomposite^7^. It is therefore very important to resolve these issues, and then fully utilise these CNTs in the polymer nanocomposite industry.

Previously, Salah *et al*.^8,9^ described a new method for producing CNTs using oil fly ash. This method, wherein ultrasonicated oil fly ash is used as a catalyst/precursor for CNT growth, seems to be promising. The CNTs are produced from a solid waste and the method has potential for mass production at a low cost. Further, the produced CNTs are short and have relatively small diameters and a zigzagged structure. Such CNTs were recently evaluated for some applications, as lubricant additives^10–12^ and for water treatment^13^. Their performance as lubricant additives was found to be superior to those of other well-known commercially available carbon nanomaterials like multi-walled CNTs, single-walled CNTs, and graphene^11,12^. On the basis of these achievements, it is of great importance to test the CNTs of oil fly ash as a reinforcing material for different polymers.

A thermoplastic is a polymer material that can be moulded at a certain temperature and then solidified by cooling. Most thermoplastics have high molecular weights and are used in a variety of products. Among them, high-density polyethylene (HDPE) has a high strength-to-density ratio, and it is widely recommended for a wide range of products owing to its high rigidity, toughness, and strength. It is produced by the polymerisation of ethylene with the aid of catalysts, mostly chromium/silicia, Ziegler–Natta, or metallocene catalysts^14^. Upon reinforcement with CNTs, this polymer might potentially be used in other applications like automobiles, aerospace, ships, sport equipment, and textiles^15^. There are several reports on the production of HDPE/CNT nanocomposites^16–21^. Some of these nanocomposites were investigated for their mechanical properties^17,19–21^. Considerable mechanical enhancements were reported, however, as mentioned above, the price of CNTs is still high and CNTs pose issues related to their agglomeration and poor interfacial bonding, which limit the commercial production of PE/CNT nanocomposites. Moreover, it is reported that the CNTs were observed to be rejected by the polymer molecules and aggregated as black bands. They were being rejected as the polymer expended energy during tensile straining^21^.

In this work, CNTs grown on oil fly ash were tested as a reinforcing material for HDPE. Xylene was used as a solvent to dissolve HDPE and to uniformly disperse CNTs. Various weight fractions of these CNTs within the range 0.5–5 wt.% were loaded into the HDPE and the mechanical properties were investigated in more details. The nanoindentation test was also performed. Other thermoplastic polymers, viz., polycarbonate, polypropylene, and polystyrene, were also preliminarily evaluated after reinforcing with 1 wt.% CNTs. The tensile strength, Young’s modulus and other mechanical properties were studied. The result of this work might be useful to suggest the use of CNTs of the solid waste oil fly ash as a proper reinforcing material for different thermoplastics polymers.

## Materials and Methods

### Materials

CNTs of oil fly ash were produced according to the method reported previously by Salah *et al*.^8,9^ while the commercial MWCNTs were purchased from Ad-Nano Technologies (Karnataka, India). HDPE was manufactured by SABIC, Saudi Arabia (Grade code B4660, medium molecular weight, Melt Flow Rate @ 190 °C & 21.6 kg load = 46 g/10 min). This polymer was reinforced by CNTs of oil fly ash at various weight fractions. Other thermoplastics from SABIC, viz., polypropylene (PP) (Grade code 57MNK10, medium molecular weight, Melt Flow Rate @ 230 °C & 2.16 kg load = 12 g/10 min), polystyrene (PS) (Grade code S100, Melt Flow Rate, 230 °C/2.16 kgf = 14 g/10 min), and polycarbonate (PC) (Grade code CFR5630, Melt Temperature = 290–310 °C, Glow Wire Flammability Index, 3.0 mm = 960 °C) were also evaluated for their reinforcement by CNTs of oil fly ash at a typical weight fraction of 1 wt.%. The polymers were used without any further processing or treatment. The CNTs powder sample was characterised using scanning electron microscopy (SEM) JSM-7500 F, field emission transmission electron microscope (FETEM) JEM 2100 F (JEOL, Japan), Raman spectroscopy (DXR Thermo scientific, USA) and Simultaneous Thermo Analyzer (STA)/Thermogravimetry (TG) and Differential Scanning Calorimetry (DSC), STA 449F3 (NETZSCH, Germany).

### Synthesis of the nanocomposites

Synthesis of HDPE/CNT nanocomposites with various weight fractions of CNTs was carried out as follows: Xylene was used to disperse the CNTs and to dissolve the PE. Initially, the desired weight fraction of the produced CNTs of oil fly ash (in the range of 0.5 to 5 wt.%) was dispersed in a small amount of xylene (20 mL) using a magnetic stirrer for 30 min, followed by sonication for 60 min. The obtained suspension was then diluted with an additional volume of xylene (~200 mL) and stirred for 30 min. Thereafter, the solution was heated to 250–300 °C and pieces of PE (20 gm) were gradually added and the mixture was stirred until they completely dissolved. Then, this solution was cured in a dish and dried at 80 °C for 24 h. After drying, the formed sheet was introduced in small pieces into a custom-made extruder that can melt the PE thermoplastic and generate filaments of ~2 mm diameter. Six 8-cm-long filaments were produced for evaluating the mechanical properties. The nanocomposites of other thermoplastics were also produced by the same method using xylene as the solvent, except in the case PC, for which dimethylformamide (DMF) was found to be the appropriate solvent.

### Characterisation and measurements

The nanocomposites of HDPE/CNTs were characterised by several techniques. The surface morphology and micro structure of the HDPE/CNT nanocomposites were studied by SEM and TEM. The thermal properties of HDPE/CNT nanocomposites were analysed by differential scanning calorimetry (DSC) performed on Shimadzu DSC-60 instrument, Japan. The DSC curves were recorded in the temperature range 20–200 °C at the heating rate of 10 °C/min. Raman spectra were recorded using a Micro Raman microscope (Thermo Fisher Scientific, USA) using an 8 mW, 532 nm laser as the excitation source. Fourier-transform infrared (FTIR) spectra of the nanocomposites were recorded using an FTIR spectrometer (Thermo Fisher Scientific, USA). These spectra were recorded in the range 600–3700 cm^−1^ with a number of scans of 32. These scans were averaged at a resolution of 4 cm^−1^. The mechanical properties of neat and nanocomposite samples with a gauge length of 25 mm (net length between the clamps) were evaluated using the LRX Plus Materials Testing Machine (Lloyd Instruments Ltd., UK) with a maximum load of 5 kN. The speed was fixed at 150 mm/min. The value reported for each specimen is the average of results of 6 test specimens.

The nanoindentation tests were performed using the NanoTest system of Micro Materials Ltd, Wrexhom, UK. This system is attached with atomic force microscopy (AFM) unit. The used indenter is 5 µm spherical diamond probe. The indenter contact velocity was fixed at 0.5 µm/s, while the load and unload rates are 10 mN/s. After a holding time of 30 s at maximum load of 50 mN the specimens were unloaded. The specimens were mounted on metallic sample holders using superglue. The NanoTest data analysis software was used to produces values of the maximum depth, plastic depth together with the derived indentation hardness. The stiffness was calculated using the linear fit method, where a tangent to the unloading curve at the maximum load is used. The average value of 5 indents (at the same load) were taken.

## Results and Discussion

**Figure 1:**
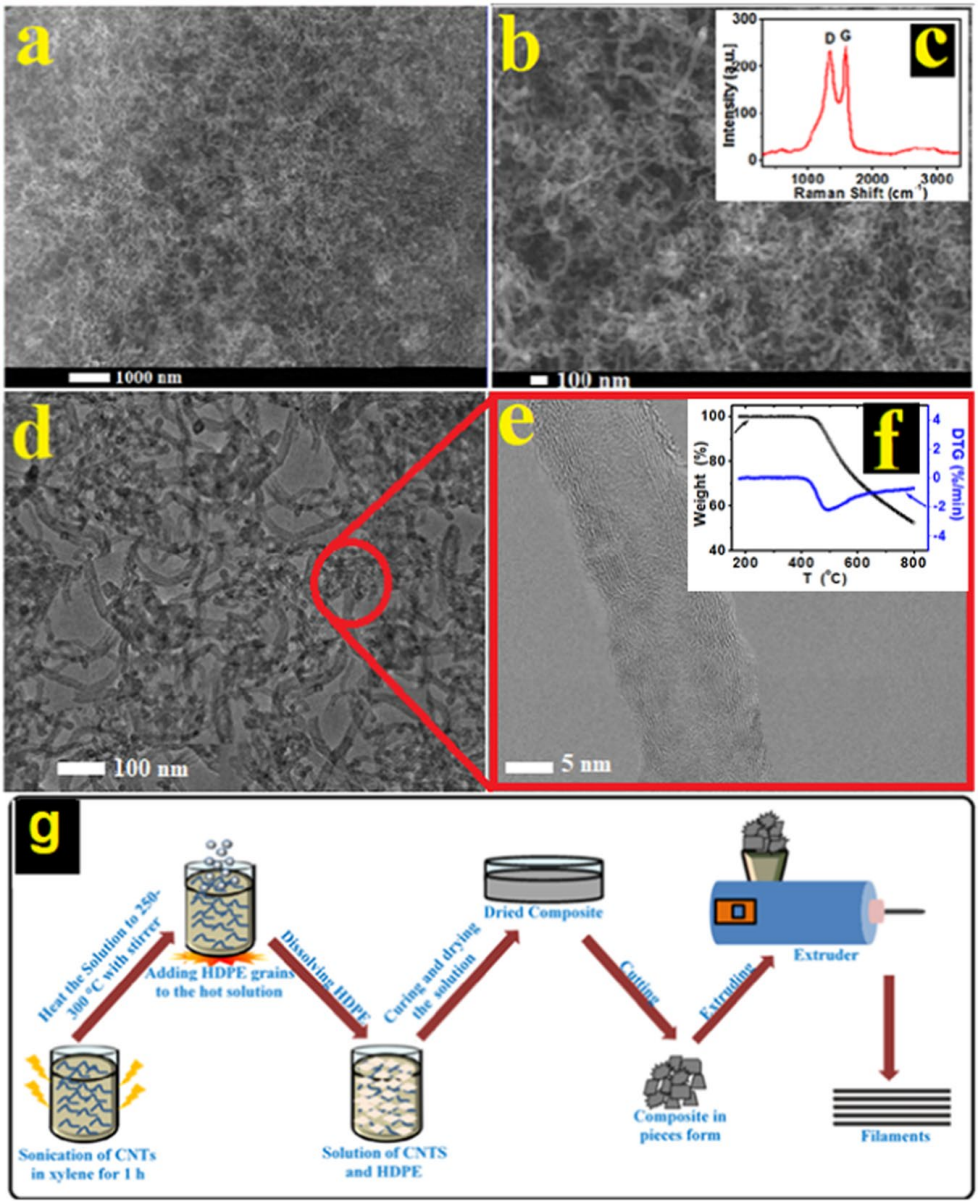
SEM images (**a** and **b**) and Raman spectrum (**c**) of CNTs of oil fly ash. TEM (**d**) and HRTEM (**e**) images are also shown. TG and DTG curves of these CNTs are shown in (**f**). An illustration of the process of producing HDPE/CNT nanocomposites is shown in (**g**).

The CNTs of oil fly ash used in this work were produced by the method described previously by Salah *et al*.^8,9^. without any modification. Thereafter, to confirm their morphology and structure, the produced CNTs were briefly investigated by SEM, TEM and Raman spectroscopy. The morphology of the CNTs in the SEM images and their bands in the Raman spectrum presented in Fig. 1(a–c) are in accordance with those reported in the previous work. Nanotubes of multiwall (around 10 walls) with a small diameter (20–30 nm) and zigzagged structure can be observed by TEM and HRTEM images Fig. 1(d,e). Raman spectrum shows the well-known graphitic (G) and defect (D) bands located at the expected positions. Further, the D/G band ratio is almost similar to that reported earlier^8,9^. Thermogravimetry (TG) and derivative thermogravimetry (DTG) curves for the produced unpurified CNTs were recorded at a heating rate of 10 °C/min and are presented in Fig. 1(f). The TG curve shows thermal stability for the produced CNTs up to around 450 °C and above this temperature the process of decomposition starts moderately fast. The DTG curve shows clear oxidation peak within the temperature range 450–520 °C. These results are in accordance with the literature data for unpurified CNTs^22^. This temperature suitability of the as produced CNTs of oil fly ash is useful to reserve the oxide groups attached on their surfaces^8,9^. These groups are quite important to assist CNTs incorporation in polymer matrices. The unique structure of these CNTs produced from solid waste oil fly ash have not been evaluated as reinforcing materials for enhancing the mechanical properties of well-known thermoplastic polymers.

We selected HDPE as the test polymer to evaluate the effect of reinforcement with CNTs of oil fly ash on its mechanical properties. Figure 1d shows the procedure adopted to produce HDPE/CNT nanocomposites. Xylene was found to be a good solvent for dissolving HDPE, and it was therefore used to initially disperse the nanomaterial, CNTs. This solvent could assist the separation of the agglomerated/entangled CNTs and improve their dispersibility in the PE matrix, and thus enable their uniform distribution in the HDPE matrix. This procedure was completed by producing uniform filaments used for the evaluation of the mechanical properties. Nanocomposites of other thermoplastics, polycarbonate, polystyrene, and polypropylene, were also produced by the same procedure using xylene as the solvent, except in the case of PC, for which DMF was used.

**Figure 2:**
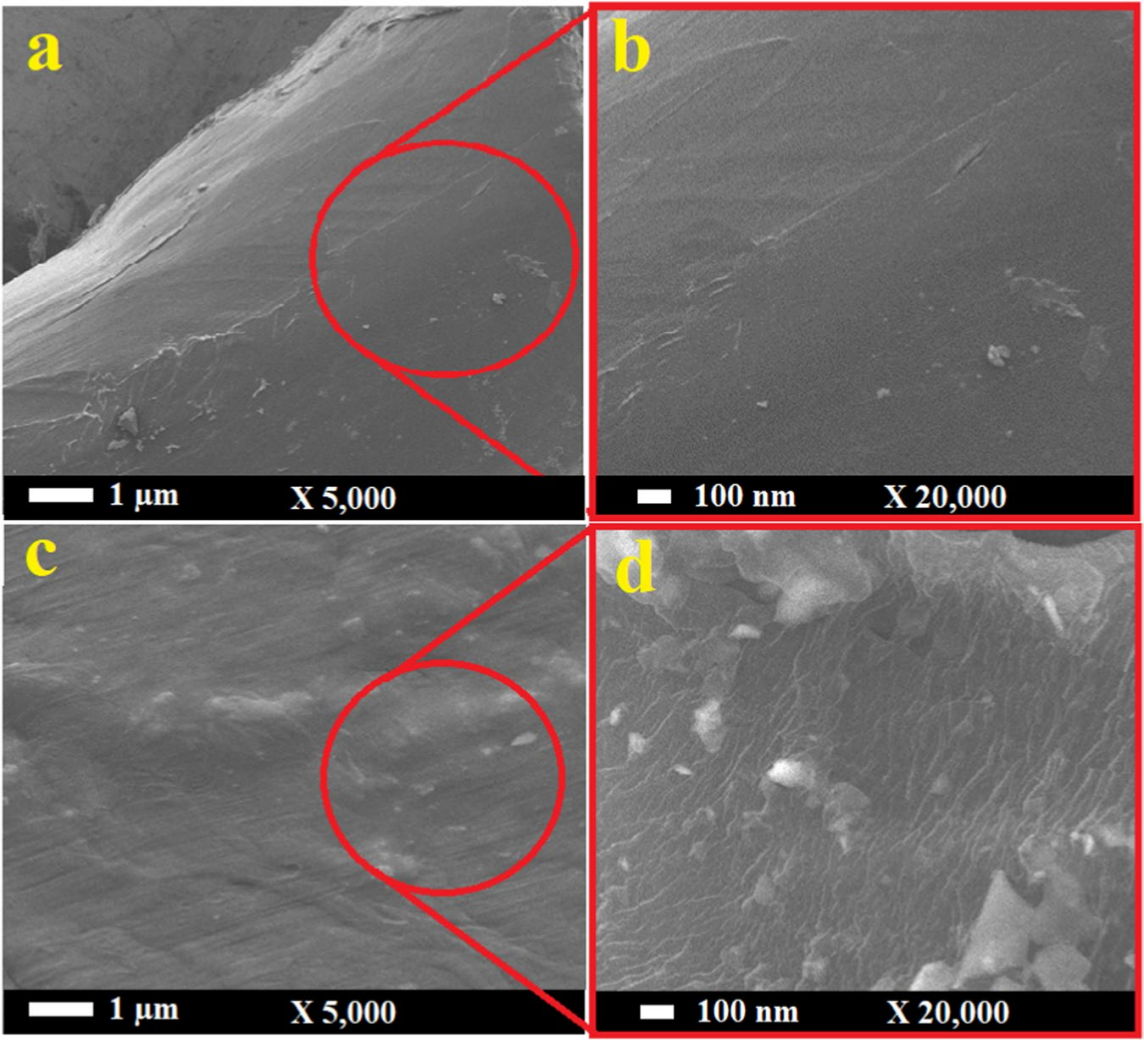
SEM images of neat HDPE (**a**,**b**) and HDPE reinforced with 3 wt.% CNTs of oil fly ash (**c**,**d**) at two different magnifications.

The surface morphology of a typical HDPE/CNT nanocomposite was investigated to observe the distribution of CNTs within the HDPE matrix. Figure 2 shows typical SEM images of neat (a and b) and PE reinforced with 3 wt.% CNTs of oil fly ash (c and d) at two different magnifications. The neat sample has a clear smooth surface with no cracks or dislocations, while the sample reinforced with 3 wt.% CNTs has different morphology. The embedded CNTs can be clearly observed; they were easily visible in the SEM images owing to their high concentration.

**Figure 3:**
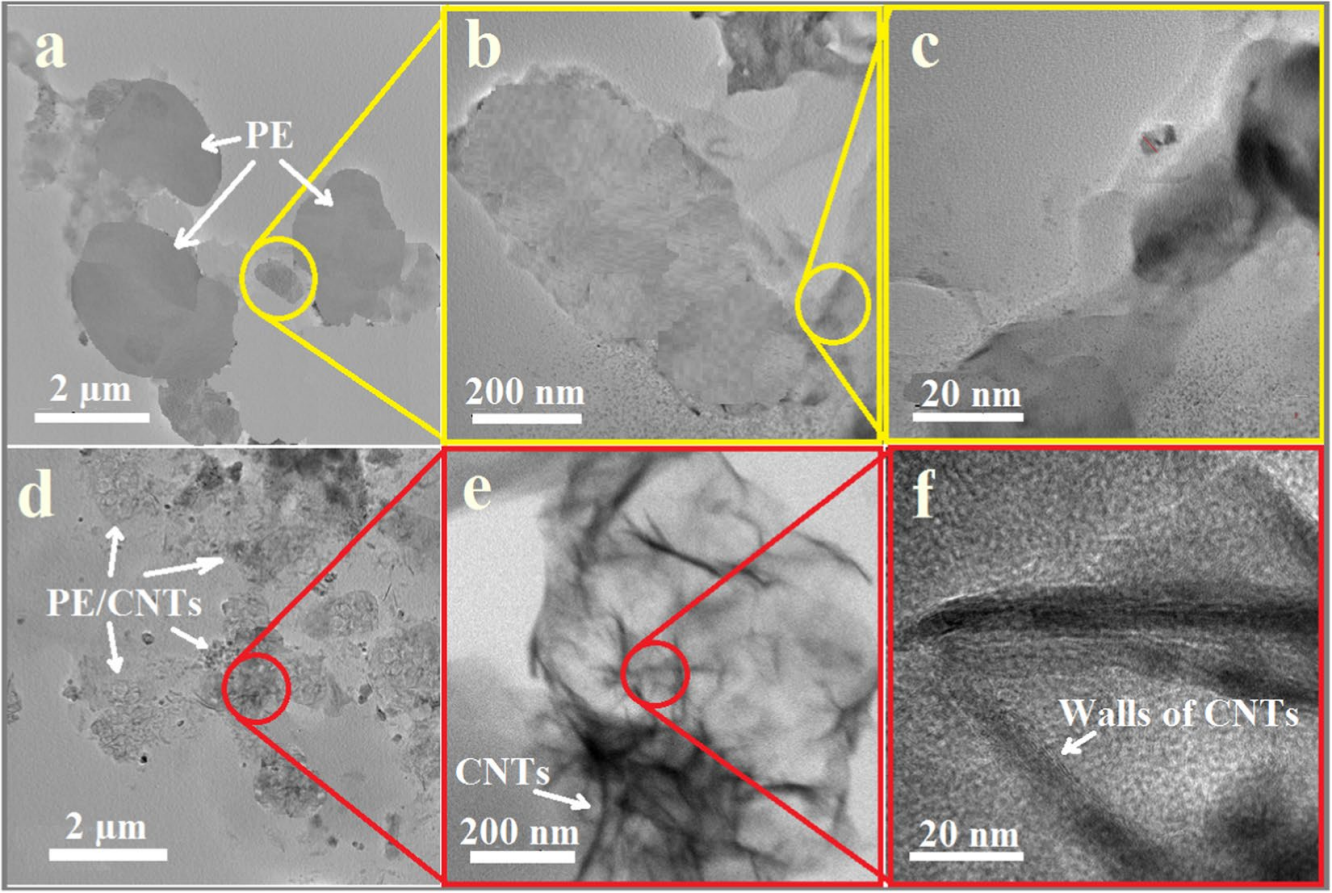
TEM images of neat HDPE (**a**–**c**) and HDPE reinforced with 1 wt.% CNTs of oil fly ash (**d**–**f**) at three different magnifications.

Distribution of CNTs in the host matrix was further confirmed by the TEM images in Fig. 3, which show the microstructure of neat HDPE (a–c) and HDPE reinforced with 1 wt% CNTs of oil fly ash (d–f). The distribution of the CNTs incorporated in the HDPE matrix seems to be uniform. This might be due to the use of a solvent for dispersing the CNTs of oil fly ash. It is well known that the distribution of carbon nanomaterials, either nanotubes or graphene, is a critical factor that can significantly affect the mechanical performance of a polymer composite. In a previous report, Bhattacharya stated that “homogeneous dispersion of the nanofiller in the polymeric matrix and strong interaction between the filler and the polymer is absolutely necessary for good reinforcement”^23^. Several approaches where suggested for proper dispersions of the nanofillers in the polymer matrix. For example, Tang and his group^24–26^ could improve the fillers dispersion by using the ball milling technique for the blend of carbon materials and polymers. They also added 3D powdered rubber nanoparticles in some of their work to improve the mechanical properties. Their approach seems to be excellent and might be modified to be applicable for thermoplastic polymers. In the present work, the first requirement, that is, a good homogeneous dispersion of the nanofiller in the polymer matrix, was successfully achieved up to some limit (as observed in the SEM and TEM images) and the promising approaches described by Tang and his group^24–26^ can be tried in further work.

**Figure 4:**
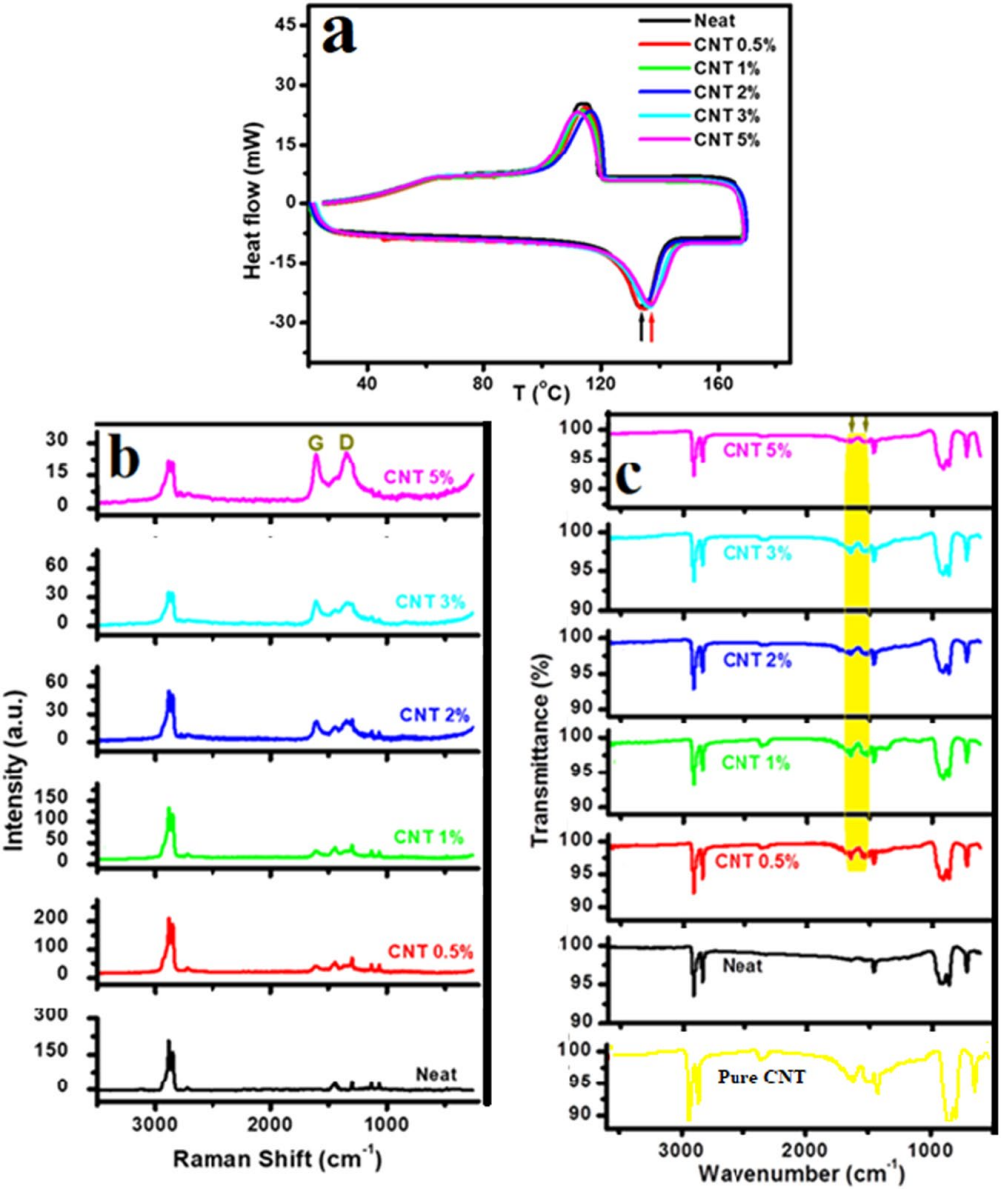
(**a**) DSC thermograms, (**b**) Raman and (**c**) FTIR spectra of HDPE/CNT polymer nanocomposites reinforced with different weight fractions of CNTs of oil fly ash.

The thermal properties of HDPE were studied to address the effect of CNTs of oil fly ash as an additive/reinforcing material. DSC thermograms of HDPE/CNT polymer nanocomposite reinforced with various weight fractions of CNTs are presented in Fig. 4a. The heating/cooling profiles of neat as well as CNT-reinforced HDPE specimens with various CNT loadings are shown in this figure. Clear endothermic peaks located at the same position can be observed in the heating curves. The crystallisation/melting point of neat HDPE can be observed at ~134 °C, while the onset temperature of melting and crystallisation is observed at ~119 °C. Small systematic increases by around 3 °C can be observed in the crystallisation temperature value after the addition of CNTs, which might be attributed to the enhanced interactions between these CNTs with polymer chins. Upon cooling, the crystallisation temperature for all the samples, the nanocomposite samples and neat HDPE, is observed at ~113 °C. The result for the neat sample is in accordance with that reported previously^27^. It is remarkable that the CNTs of oil fly ash do not have a considerable negative effect on the thermal properties of HDPE.

Raman spectra of the HDPE/CNT nanocomposite reinforced with various weight fractions of CNTs of oil fly ash are presented in Fig. 4b. The main bands of neat HDPE are observed at their regular positions. The band at 1440 cm^−1^ is the –CH_2_ bending mode, which is an indicator of the crystalline HDPE phase. Normally, HDPE has a higher degree of crystallinity^28,29^. The symmetric CH_2_ stretching modes of HDPE can be observed at 2848 and 2882 cm^−1^. The intensity of the first band is slightly lower than that of the second one, which also indicates higher crystallinity. The bands at 1065, 1130, and 1300 cm^−1^ correspond to C–C anti-symmetric stretching, symmetric stretching, and twisting modes^30^. In case of the HDPE nanocomposites incorporated with CNTs, the HDPE bands still dominate, although their intensity has decreased. These bands are accompanied by two new bands located at 1345 and 1598 cm^−1^. These new bands are the defect (D) and graphitic (G) vibrations of CNTs^8,9^ (see Fig. 1c). Their intensity increased upon increasing the concentration of CNTs in the HDPE matrix. A noticeable change in the PE structure with the addition of CNTs is observed in the band intensities along with a change in the intensity ratio between the 2848 and 2882 cm^−1^ bands. The intensity of the first band is found to increase slightly compared to that of the second one. This indicates a slight reduction in the crystallinity of HDPE.

FTIR spectra of HDPE/CNT nanocomposites reinforced with various weight fractions of CNTs of oil fly ash are presented in Fig. 4c. The spectrum of neat HDPE shows regular bands, which are the stretching vibrations of –CH_2_ at 2950–2850 cm^−1^, the bending vibrations of –CH_2_ at 1470 cm^−1^, and the rocking vibration of –CH_2_ at 720 cm^−1^ ^31^. Two crowded/overlapping bands can also be observed at ~870 and 940 cm^−1^. These bands might be due to the presence of some catalyst used in the production of HDPE, which are often chromium/silicia, Ziegler–Natta, or metallocene catalysts^14^. Crowded bands were also observed in some products of HDPE. The bands observed by Saker *et al*.^32^ in the fractional fuel produced from HDPE at 888.28 and 965.09 cm^−1^ were attributed to the –C=CH_2_ and –CH=CH–(trans) functional groups, respectively. The present HDPE might have these functional groups, and therefore shows these two bands at 870 and 940 cm^−1^. In case of HDPE/CNT nanocomposites, the FTIR spectra almost resemble that of neat PE, except for the emergence of two small bands located at 1540 and 1640 cm^−1^ (shown in the yellow shadow area of Fig. 4c). These bands of pure CNTs of oil fly ash. The first one might be ascribed to the carboxylate/carboxylic acid groups present in the used CNTs of oil fly ash^9^, which perhaps could form bonds between the CNTs and HDPE. This band was also observed in oxidised multi-walled carbon nanotubes produced by Deng *et al*.^33^. The second band at 1640 cm^−1^ (Fig. 4c) of the pure nanotubes is due to the C=C stretching of these CNTs^8^.

The mechanical properties of the HDPE reinforced with different weight fractions of CNTs of oil fly ash are presented in Table 1. These properties include the tensile strength, stiffness, Young’s Modulus, load at break, stress at break, hardness, and elongation at fracture. It is clear that the optimum weight fraction of CNTs at which all the aforementioned mechanical properties of the nanocomposite are considerably enhanced is between 1 and 2 wt.%., except the elongation at fracture, which decreased with the introduction of CNTs, even at a low concentration. The elongation at fracture values given in Table 1 have high standard deviation values. The mechanical properties of the HDPE reinforced with 1 wt.% of a commercial MWCNTs were also included in this work. The observed enhancements are close to those of the 1 wt% CNTs reinforced HDPE. These enhancements by the commercial MWCNTs are even lower, particularly the tensile strength, stiffness and load at break, which indicates a superiority for the CNTs of oil fly ash as proper reinforcements.

**Table 1:** Mechanical properties of HDPE reinforced with various weight fractions of carbon nanotubes (CNTs) of oil fly ash.

	Neat PE	PE/CNT 0.5%	PE/CNT 1%	PE/CNT 2%	PE/CNT 3%	PE/CNT 5%	PE/MWCNT 1% (Commercial)
Tensile Strength (MPa) (±8%)	26.52	28.42	29.61	31.928	25.71	23.58	28.69
Stiffness (N/m) (±7%)	57703	62068	65755	64765	43293	44330	62880
Young’s Modulus (MPa) (±12%)	468.18	533.05	586.79	647.30	367.39	363.45	591.79
Load at Break (N) (±10%)	48.31	50.50	51.97	44.62	38.14	43.85	50.07
Stress at Break (MPa) (±8%)	15.92	17.08	17.29	18.01	14.25	14.57	17.61
Hardness (Shore D) (±10%)	80.47	81.04	84.20	81.12	68.83	71.57	83.20
Elongation at Fracture (mm) (±25%)	202.04	138.29	118.11	102.54	64.85	23.86	110.11

**Figure 5:**
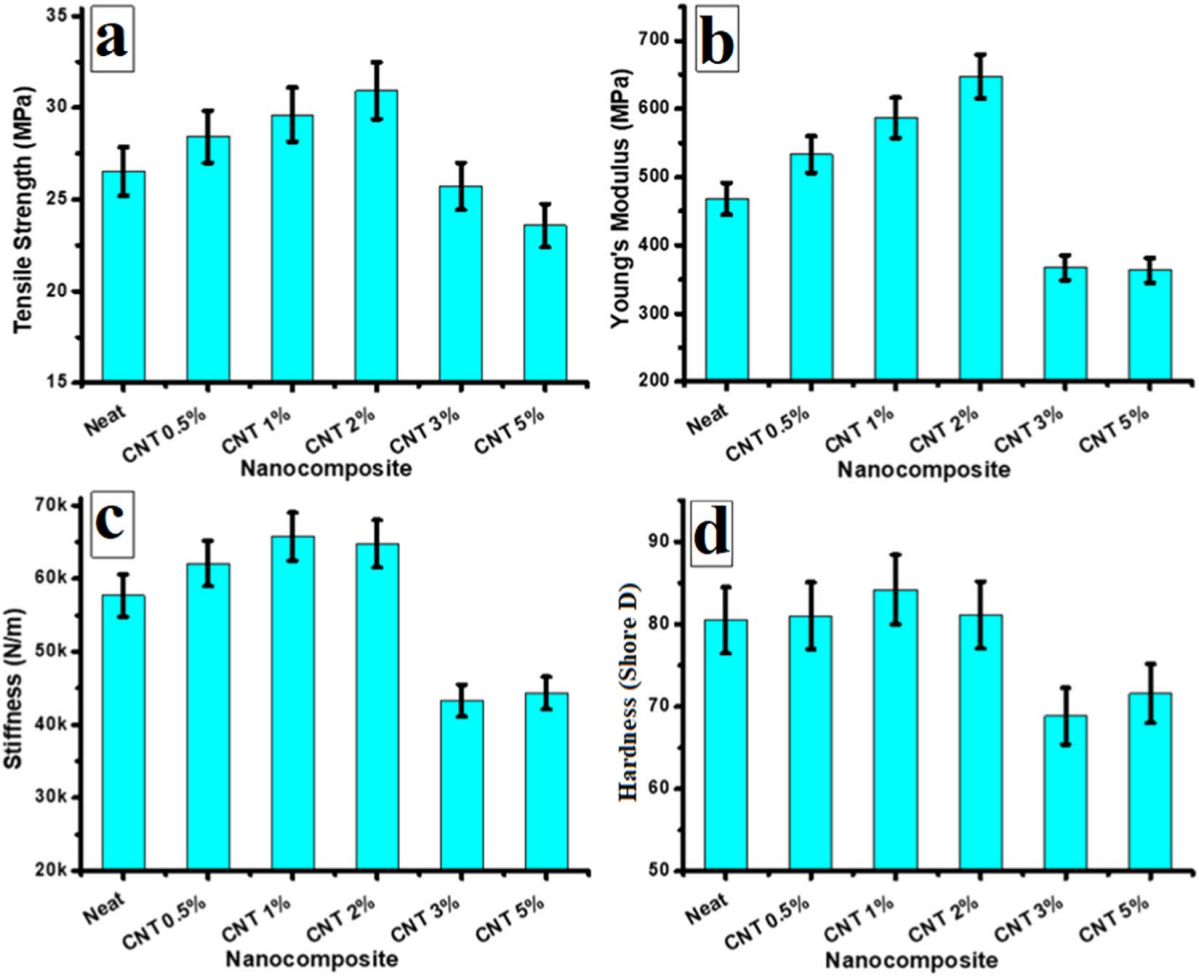
Mechanical properties of HDPE/CNT polymer nanocomposites reinforced with different weight fractions of CNTs of oil fly ash. (**a**) Tensile strength, (**b**) Young’s Modulus, (**c**) stiffness and (**d**) hardness.

Some of the investigated properties presented in the above table are also plotted in Fig. 5a–d. This figure shows that for maximum enhancement of the tensile strength (Fig. 5a), the CNT concentration should be 2 wt.%. At this concentration of CNTs, the tensile strength of the HDPE/CNT nanocomposite is increased by ~20.4% compared to that of neat HDPE. The major effect of reinforcing with CNTs was observed in the Young’s Modulus, which was found to be ~38% higher compared to that of neat HDPE (Fig. 5b). The stiffness and hardness also increased by ~14 and 5%, respectively than those of neat HDPE (Fig. 5c,d). The used CNTs of oil fly ash have been previously reported^9^ to have –COO and –COOH groups, which might contribute to the binding of CNTs to the polymer chains at optimum concentrations of the CNT, thereby increasing the mechanical properties of HDPE. But, at higher concentrations of CNTs, e.g. 3 and 5 wt%, a drastic decline in all these mechanical properties occurred, with the values of the various parameters being lower than those of the neat HDPE sample. This decrease in the mechanical properties at high content of CNTs (e.g. above 2 wt.%) might be attributed to the formation of clusters or agglomerates. These clusters could act as defects and serve as points of crack initiation.

**Figure 6:**
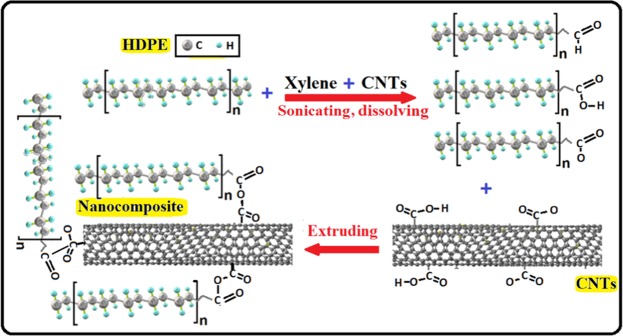
Schematic illustration of the possible interaction between HDPE and CNTs of oil fly ash.

As mentioned above, xylene was used as a solvent to disperse the CNTs and also to dissolve the HDPE pieces, therefore the HDPE chains will be broken and carbon free radicals might be generated. In the meantime, some of the oxygenous functional groups such as carboxylic group (–COOH) and hydroxyl group (–OH) have been confirmed by the FTIR and XPS studies reported earlier in the CNTs of oil fly ash^9,13^. These groups might also be attached to the broken HDPE chains during the sonication and dissolving process. Therefore, it is possible for these attached and generated radicals to further react with the remaining radical groups in CNTs and form new bonds through covalent and non-covalent bonding. Some of these new bonds are clearly observed in the FTIR results presented in Fig. 4c. Other carbonyl groups might also be formed meanly during the filaments formation as a result of oxygen and carbon dioxide reactions in air. This kind of reaction, which results on carbonyl groups formation has been reported to introduce these radicals into the polyethylene chains^34^. These views are illustrated in Fig. 6.

**Figure 7:**
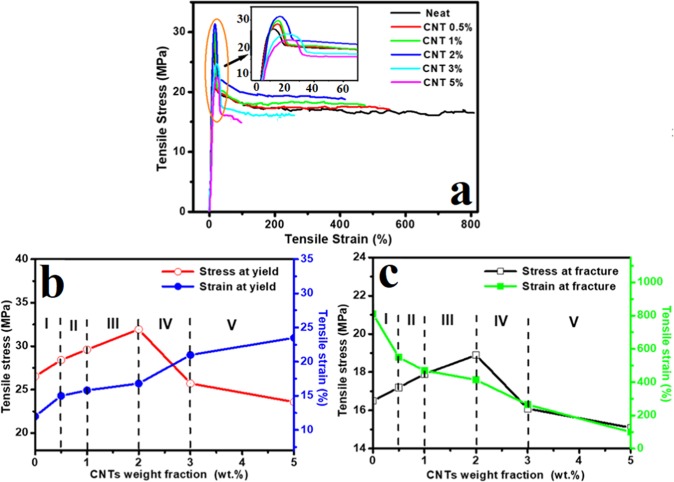
(**a**) Stress-strain curves of HDPE/CNT polymer nanocomposites reinforced with different weight fractions of CNTs of oil fly ash. Influence of the CNTs content on the tensile stress and strain at (**b**) yield point and (**c**) fracture of the HDPE/CNT nanocomposites.

The stress-strain curves of HDPE/CNT polymer nanocomposites reinforced with various weight frication of CNTs of oil fly ash are presented in Fig. 7a. The neat sample showed ultimate stress (at the yield point) of ~26.5 MPa, and beyond the ultimate stress, the filament started to form a neck and then the load fell until the sample broke. A maximum value of ~800% was recorded for the tensile strain. This strain for neat HDPE is close to that reported by others^35^. The features of the curve also resemble those reported previously for the stress-strain curve of HDPE^36^. The tensile strain decreases with increasing CNT concentration, reaching ~100% at 5 wt.% loading of CNTs. This might be due to the presence of CNTs, which has been reported to have a negative effect on the elongation at break point^20^. CNTs were also found to restrict the mobility of the HDPE chains and improve the tensile strain hardening effect during the deformation of the polymer^37^, leading to increased ultimate stress values. It is also clear that, with the addition of CNTs to the host matrix of HDPE, the above optimum value of the yield point decreased and slightly shifted to a higher strain value (see inset of Fig. 7a). Slight broadening of the peak of the yield point with increasing CNT weight fraction can also be observed.

Figure 7 shows the influence of the CNTs weight fraction on the tensile stress and strain at the yield point (b), and fracture (c) of the HDPE/CNT nanocomposites. Both Fig. 7b,c were sectioned into five zones according to the CNTs content within the HDPE polymer host. In zone I of Fig. 7b, corresponding to 0–0.5 wt.% CNT content, a small increase in both the tensile stress at yield and tensile strain of HDPE/CNT nanocomposites is observed. The rate of increase in the tensile strain is higher than that of the tensile stress at the yield point. This improvement might be attributed to the formation of a number of bonds between the sites of HDPE matrix and CNTs. The surface area per unit volume is also high for CNTs, which allows a good interface with HDPE sites. This will also make a good network for load transfer, where the stress is distributed. This behaviour is almost continued in zones II (0.5–1 wt.%) and III (1–2 wt.%), but at lower rate in the case of tensile strain. At the end of zone III e.g. close to 2 wt.% of CNTs, the stress at yield is considerably high; at this point, the number of links between the CNTs and HDPE matrix would be maximised. A further increase in the CNT content (zones IV and V) leads to further increase in the tensile strain, but with a considerable decrease in the yield stress. This might be due to the formation of clusters or agglomerates above 2 wt.% of CNTs. These clusters could act as defects and serve as points of crack initiation, thereby limiting the tensile stress at yield. However, high concentrations of CNTs do not affect the tensile strain value; it increased continuously with increasing CNT content, probably due to numerous bonds formed between the CNTs and HDPE sites.

The effect of the added CNTs on the stress at fracture and strain at fracture of the nanocomposites are shown in Fig. 7c. It is obvious that at low weight fractions (zones I, II and III), CNTs have a clear positive effect on the stress at fracture; however, beyond these zones, CNTs have a negative effect (zones IV and V). This behaviour is similar to that of the stress at yield shown in Fig. 7b. The negative effect at higher concentrations of CNTs might be due to the presence of CNTs agglomerates in the HDPE/CNT nanocomposites. Such agglomerates might act as initiation sites for the generation and extension of cracks^38,39^. On the other hand, the effect of CNTs on the strain at fracture is negative in the whole range of CNTs weight fractions (zones I, II, III, IV and V), which is completely opposite to that of the strain at yield shown in Fig. 7b. The reduction in strain at fracture of HDPE/CNT nanocomposites may be attributed to the power of CNTs for hindering the crack propagation to resist the improvement in strain. This power might not be enough to efficiently hinder the crack propagation within the host. The other possible reason is that CNTs can absorb the fracture energy through interfacial deformation between the CNTs and HDPE matrix such as debonding or pullout^35^. The aforementioned results of stress at yield and stress at fracture (Fig. 7b,c) reveal that the CNT additive acts as a reinforcement material in zones I, II, and III, but causes defects in zones IV and V. In the cases of strain at yield and strain at fracture, CNT addition can enhance the former and reduce the latter.

**Figure 8:**
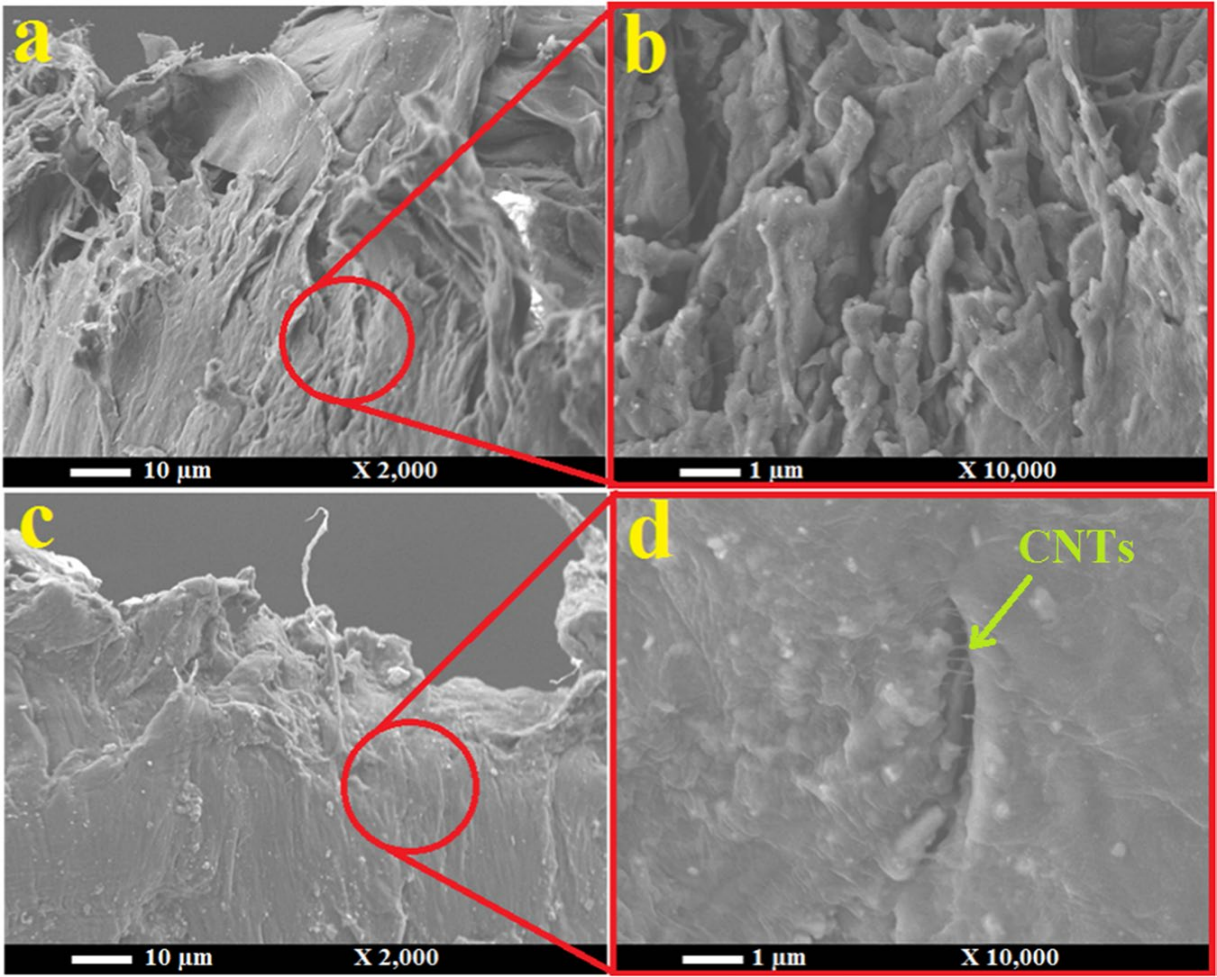
SEM images of the fractured areas of the neat HDPE (**a,b**) and HDPE/CNT nanocomposite with 1 wt.% CNT (**c,d**).

The fractured areas of neat HDPE and the HDPE/CNT nanocomposite were investigated by SEM observations at different magnifications. The SEM images presented in Fig. 8(a,b) show the fracture of neat HDPE. It is clear that large fragments and robe-like structures are formed as a result of the applied load. These indicate that the parts/sites of neat HDPE tend to easily disassemble from each other or decompose, but have a good ability to extend. In the case of HDPE/CNT nanocomposite sample shown in Fig. 8c,d, these sites/parts are well-bonded with each other, without forming robe-like structures, which indicates that CNTs could effectively join these sites, and act as crack bridging even at the fracture areas. Some CNTs joining these sites around a crack can be observed in image d, which is a great advantage of these tiny entities in having a significant impact on the microstructure and load transfer in these polymers, and thus their mechanical properties^21^. The appearance of individual CNTs confirms the disentanglement and accumulation of CNTs during tensile loading. The nanofillers and polymers interfacing is the key point to enhance the mechanical properties of the resulting nanocomposites. Several approaches were described in the literature to improve the nanofillers dispersion and their interfacing with the polymer sites^40–42^. Surfactants treatments for the nanofillers is one of the suggested methods, which has been found to be effective.

**Figure 9:**
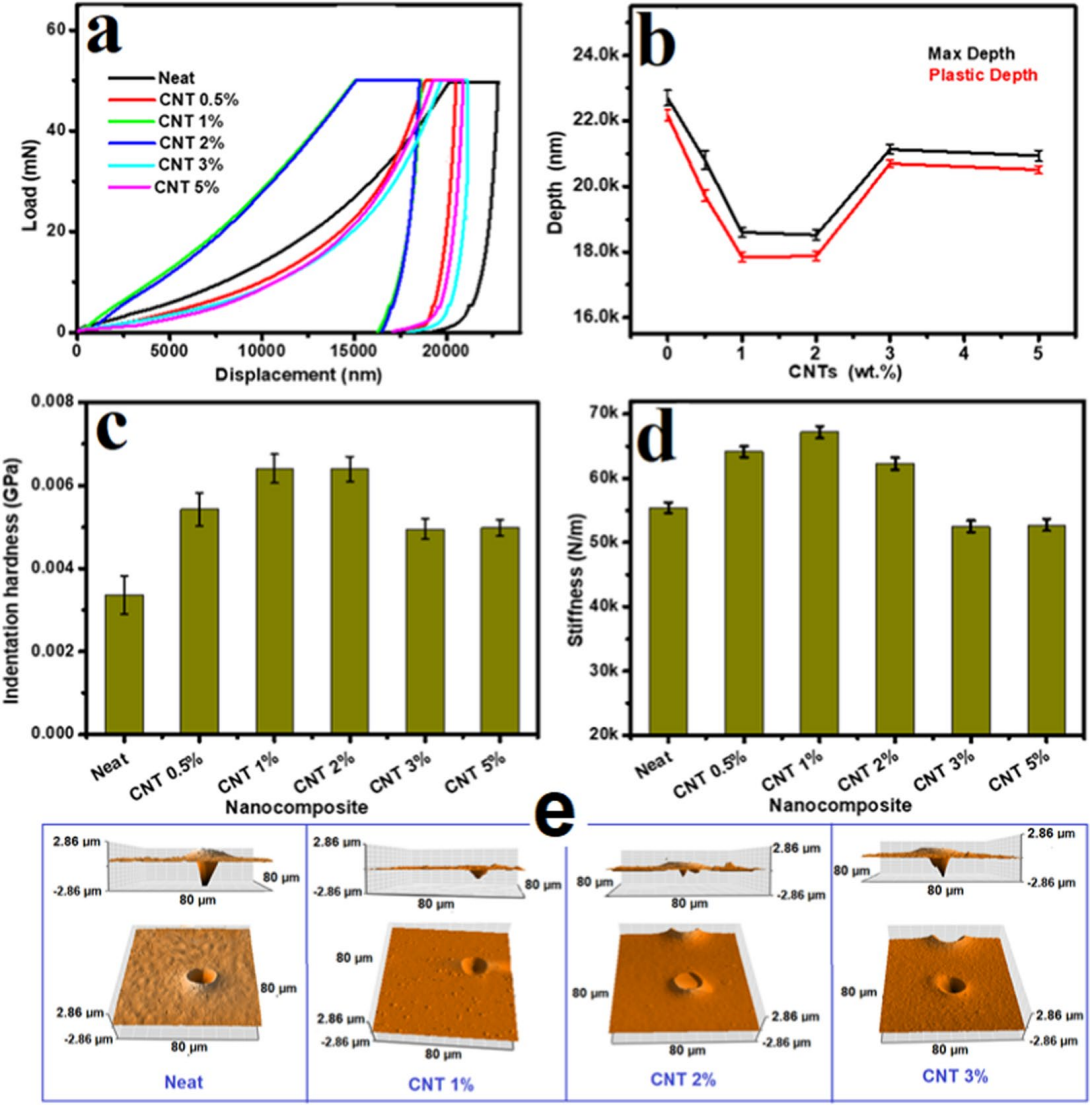
Nanoindentation properties of HDPE/CNT polymer nanocomposites reinforced with different weight fractions of CNTs of oil fly ash: (**a**) Load and unload displacement curves, (**b**) Maximum and plastic depths, (**c**) Indentation hardness, and (**d**) Stiffness. (**e**) Three-dimensional and height profile AFM images of the nanoindentation pits after performing the test at 50 mN load on the neat HDPE and HDPE/CNT nanocomposites of 1, 2 and 3 wt.% CNTs loading.

To get more insights about the mechanical properties of HDPE/CNT nanocomposites, the nanoindentation tests were performed and the obtained results are presented in Fig. 9. This figure shows the load-displacement curves (a), maximum and plastic depths (b), indentation hardness (c) and stiffness (d) for the pure and CNTs loaded HDPE. Three-dimensional AFM images with height profile of the nanoindentation pits after performing the test at 50 mN load of the neat HDPE and HDPE/CNT nanocomposites with 1, 2 and 3 wt.% CNT were also obtained and presented in Fig. 9e. The maximum and plastic depths were significantly decreased in the nanocomposite samples, particularly with CNTs loading in the range 1–2 wt.% (Fig. 9b). This lower displacement might be attributed to the higher resistance offered by the HDPE matrix incorporated with hard CNTs to the indenter. At higher load of CNTs e.g. above 2 wt%, it is possible that the nanocomposite will have some amorphous phases, where the HDPE crystallinity was observed to decrease (Fig. 4).

The indentation hardness values were also observed to be maximum in the nanocomposites of 1–2 wt.% CNTs loading (Fig. 9c). Around 63% increases in the indentation hardness values is observed at 1–2 wt% of CNTs loading. The presence of hard reinforcement CNTs increases the load bearing capacity of the pure HDPE matrix and limit the matrix deformation by confining movement of dislocation. These results showed clearly the hardness enhancements achieved by including CNTs into the host of HDPE. Measurements of indentation hardness for HDPE were reported in the literature by many groups. They found large variations in the obtained values of indentation hardness^43,44^. This might be due to the use of different techniques and conditions to calculate the indentation hardness in MPa or in GPa. It has been reported that hardness is not always an intrinsic property of a material^45^ and the obtained values are due to a complex combination of deformation and elastic behavior. Nevertheless, the obtained results showed that CNTs are effective enhancers for this thermoplastic material. The stiffness values were also observed to be higher in the nanocomposite samples with a maximum value achieved at 1 wt% CNTs loading (Fig. 9d). This result is almost similar to that presented above using the tensile method (Fig. 7a). These improvements can be observed clearly in Fig. 9e. This figure shows three-dimensional and height profile AFM images of the nanoindentation pits after performing the test at 50 mN load on the neat HDPE and HDPE/CNT nanocomposites of 1, 2 and 3 wt.% CNTs loading. It is clear that the depths of the nanoindentation pits are lower in the 1 and 2 wt% CNTs loaded nanocomposite samples. The pits are deeper in case of the neat and 3 wt% CNTs loaded samples. The formation of amorphous phases at higher load of CNTs, might be the reason behind the observed decrease in the hardness and stiffness of HDPE.

The mechanical properties of other polymers, PC, PP, and PS, reinforced with 1 wt.% of CNTs of oil fly ash are presented in Table 2. The studied mechanical parameters are the tensile strength, stiffness, Young’s modulus, load at break, stress at break, hardness, and elongation at fracture. These preliminary results show clear enhancement of most of these properties. The mechanical properties of PC improved significantly upon adding 1 wt.% of CNTs of oil fly ash. The improvements are observed mainly in the tensile strength, stiffness, Young’s modulus, load at break, stress at break, and hardness. The mechanical properties of PS, mainly its tensile strength, Young’s modulus, load at break, stress at break, hardness, and elongation at fracture, could also be improved. The PS/CNT nanocomposite showed relatively lesser improvement in the mechanical properties, and even reduction in some of these properties, as compared to those of nanocomposites of HDPE, PC, and PS with CNTs. Incorporation of CNTs in the matrix of these polymers has been studied previously^46–48^. These preliminary results obtained mainly for PC and PS need to be investigated further in more detail by loading the polymers with different weight fractions of CNTs of oil fly ash.

**Table 2:** Mechanical properties of polycarbonate (PC), polypropylene (PP), and polystyrene (PS) reinforced with 1 wt.% CNTs derived from oil fly ash.

	Neat, PC	PC/CNTs 1%	Neat, PP	PP/CNTs 1%	Neat, PS	PS/CNTs 1%
Tensile Strength (MPa) (±8%)	35.907	43.556	25.275	26.368	38.42	44.762
Stiffness (N/m) (±7%)	69170	70403	49583	43463	72756	61231
Young’s Modulus (MPa) (±12%)	1088.7	1208.8	635.84	646.56	1178.6	1267.3
Load at Break (N) (±10%)	48.552	72.546	59.584	53.192	55.773	74.040
Stress at Break (MPa) (±8%)	20.859	32.372	19.972	21.208	27.930	36.816
Hardness (Shore D) (±10%)	83.7	99.206	75.459	66.391	86.859	90.54
Elongation at Fracture (mm) (±25%)	9.4419	6.7	46.882	58.497	8.1297	11.76

## Conclusions

CNTs of oil fly ash were evaluated as reinforcing materials for different thermoplastics, mainly HDPE. These CNTs were easily dispersed in the host HDPE matrix, resulting in considerable improvement in the mechanical properties. Low weight fractions of these CNTs, within the range 1–2 wt.%, were found to be effective for enhancing the tensile strength and Young’s modulus by ~20 and 38%, respectively. Other mechanical properties like load at break and hardness were also improved. The nanoindentation results were also found to be in support to these findings. Additional thermoplastic polymers like polycarbonate, polypropylene, and polystyrene were also evaluated after their reinforcement with 1 wt.% CNTs. Promising results were obtained, suggesting that CNTs of the solid waste oil fly ash might be a suitable reinforcement material for different thermoplastics polymers.
